# Ribosomal proteins can hold a more accurate record of bacterial thermal adaptation compared to rRNA

**DOI:** 10.1093/nar/gkad560

**Published:** 2023-07-03

**Authors:** Antonia van den Elzen, Karla Helena-Bueno, Charlotte R Brown, Lewis I Chan, Sergey V Melnikov

**Affiliations:** Biosciences Institute, Newcastle University, Newcastle upon Tyne NE2 4HH, UK; Biosciences Institute, Newcastle University, Newcastle upon Tyne NE2 4HH, UK; Biosciences Institute, Newcastle University, Newcastle upon Tyne NE2 4HH, UK; Biosciences Institute, Newcastle University, Newcastle upon Tyne NE2 4HH, UK; Biosciences Institute, Newcastle University, Newcastle upon Tyne NE2 4HH, UK

## Abstract

Ribosomal genes are widely used as ‘molecular clocks’ to infer evolutionary relationships between species. However, their utility as ‘molecular thermometers’ for estimating optimal growth temperature of microorganisms remains uncertain. Previously, some estimations were made using the nucleotide composition of ribosomal RNA (rRNA), but the universal application of this approach was hindered by numerous outliers. In this study, we aimed to address this problem by identifying additional indicators of thermal adaptation within the sequences of ribosomal proteins. By comparing sequences from 2021 bacteria with known optimal growth temperature, we identified novel indicators among the metal-binding residues of ribosomal proteins. We found that these residues serve as conserved adaptive features for bacteria thriving above 40°C, but not at lower temperatures. Furthermore, the presence of these metal-binding residues exhibited a stronger correlation with the optimal growth temperature of bacteria compared to the commonly used correlation with the 16S rRNA GC content. And an even more accurate correlation was observed between the optimal growth temperature and the YVIWREL amino acid content within ribosomal proteins. Overall, our work suggests that ribosomal proteins contain a more accurate record of bacterial thermal adaptation compared to rRNA. This finding may simplify the analysis of unculturable and extinct species.

## Graphical Abstract

**Graphical Abstract:**
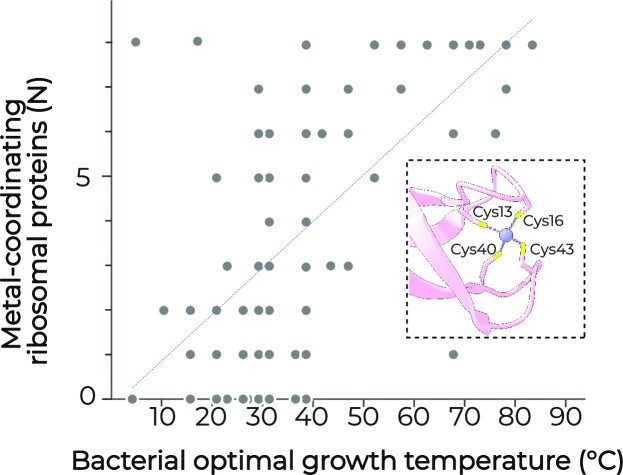

## INTRODUCTION

Genes coding for ribosomal RNA (rRNA) and ribosomal proteins are widely used as molecular clocks to infer phylogenetic relationships among species and gain insights into the origin of life (1–12). It is unclear, however, whether these genes contain other information, such as information about the optimal growth environment for a given species.

Previous studies found that ribosomal genes contain some information about a given organism’s optimal growth temperature and halotolerance (13–23). For instance, thermophilic ribosomal proteins were found to have a higher content of certain amino acids, such as arginine, isoleucine, proline and tyrosine, and a decreased content of serine and threonine. However, this correlation was not consistent across species to serve as an accurate predictor of an organism’s optimal growth temperature (14,15).

A more promising approach was established by studying the correlation between the optimal growth temperature and the GC content in rRNA (16–23). For example, an almost linear correlation was observed in 143 microbial genera, where the rRNA GC content gradually increased from ∼45% GC in psychrophilic bacteria to ∼55% GC in thermophilic bacteria (16). However, at least 22 other studied genera showed temperature-independent variations, possibly because the rRNA GC content was found to be influenced by other factors, including high-salt conditions and host-restricted lifestyles (16,24–29). As a result, several improvements have been suggested to mitigate the presence of ‘outliers’ when estimating thermal adaptation. One approach suggested to use the uracil content instead of the GC content in 16S rRNA sequences (17). Another approach suggested to use the GC content of the helical segments of the 16S rRNA (19,20). Currently, the GC content of the helical segments in rRNA serves as the most commonly used ribosome-based proxy for assessing the thermal adaptation of bacteria and archaea (30,31).

At the same time, alternative strategies for culture-independent estimation of the optimal growth temperature have been developed based on the analysis of complete genomes (32–34). One of the most accurate prediction strategies was found empirically by correlating the optimal growth temperature of microbial species with the content of seven amino acids—namely Y, V, I, W, R, E and L—within the predicted cellular proteomes (34). Collectively, these studies showed that using a single gene of rRNA as a ‘molecular thermometer’ is a promising approach but on its own it lacks accuracy, raising the question: Do ribosomal genes contain other markers of thermal adaptation that could help estimate the optimal growth conditions for a given organism based on sequencing of its most conserved genes?

Here, we addressed this question by correlating the experimentally defined growth temperatures for 2021 representative bacteria with the sequences of their ribosomal proteins.

Our first strategy was to abandon the traditional approach in which sequences of rRNA or proteins are treated as strings of equally important residues to calculate their contents. Instead, we focused our attention on a small number of residues that are critically important for the folding of ribosomal proteins in thermophiles. Specifically, studies of *Thermus thermophilus* ribosomes revealed that the smallest ribosomal proteins coordinate metal ions to enable protein folding at high temperatures: the protein uS4 coordinates an iron–sulfur cluster and the proteins uS14, bS18, uL24, bL28, bL31, bL32, bL33 and bL36 coordinate zinc ions (35–39). Furthermore, analysis of ribosomal proteins from 30 bacterial species revealed that thermophiles *Aquifex*, *Thermotoga* and *Thermus* almost always have metal-binding residues in their ribosomal proteins (39). This led to the hypothesis that these residues are adaptive and might contribute to the stability of the ribosome at high temperatures (39). However, the limited amount of sequencing data available at the time did not allow to test this hypothesis, and it remained unclear whether the metal-binding sites could serve as a better proxy for the optimal growth temperature compared to 16S rRNA.

Here, we tested whether the hypothesis that the optimal growth temperature of a living cell can be predicted using 36 metal-binding residues in ribosomal proteins can indicate the optimal growth conditions of a living cell. Taking an advantage of our recent progress in annotating bacteria by their optimal growth conditions (40), we analyzed over 24 000 sequences of ribosomal proteins from bacteria with experimentally defined optimal growth temperatures. We found that the metal-binding sites in ribosomal proteins act as adaptative features that are required for bacteria thriving above 40°C, but not in bacteria adapted to lower temperatures. We then found that the presence of these 36 metal-binding residues shows a better correlation with the optimal growth temperature compared to the currently used method based on the 16S rRNA GC content. Lastly, we determined that the most precise ribosome-based proxy among all known proxies is the content of YVIWREL amino acids within ribosomal proteins.

Collectively, our study showed that ribosomal proteins can serve as more accurate proxies for assessing bacterial thermal adaptation in comparison to the commonly used method based on the analysis of 16S rRNA. By using the metal-binding sites of ribosomal proteins, we could reduce the amount of data without reducing prediction accuracy. Alternatively, by using the YVIWREL content of ribosomal proteins, we could significantly increase prediction accuracy compared to predictions based on using 16S rRNA. Thus, our newly identified proxies of thermal adaptation provide an independent tool to estimate the optimal growth temperature of uncharacterized species, as well as the ancient history of climate change through sequencing of the ribosomal genes of extant and extinct species.

## MATERIALS AND METHODS

### Annotating bacterial species with their optimal growth temperatures

The values of experimentally determined optimal growth temperatures were scraped from 23 public repositories of microorganisms, including the ATCC, the DSMZ and others (Supplementary Table S1). The retrieved values were then added to the list of representative bacteria with fully sequenced genomes (https://www.ncbi.nlm.nih.gov/genome/browse). The resulting file (Supplementary Data 1) was then deposited in the database of organisms with experimentally defined optimal growth temperatures (http://melnikovlab.com/gshc/).

### Retrieving sequences of bacterial ribosomal proteins

To retrieve sequences of ribosomal proteins, we searched the UniProt databank for protein sequences that are named ‘30S ribosomal protein X’ or ‘50S ribosomal protein Y’, where X and Y corresponded to the bacterial name of ribosomal proteins from the small and large subunits, respectively. These data were then cleaned from truncated sequences by removing those sequences that were at least 25% shorter than the average length of a given protein in our dataset. The resulting files contained ∼2400 sequences per each ribosomal protein (Supplementary Data 2).

### Assessing temperature-associated variations in bacterial ribosomal proteins

To identify temperature-associated variations in the ribosomal proteins, we aligned protein sequences using Clustal Omega (41) with default settings (Supplementary Data 3), and then analyzed these alignments using the BioAlign package from Biopython (42). In this analysis, for each ribosomal protein, we calculated two consensus sequences: a consensus sequence for cold-adapted bacteria (adapted to growth below 20°C) and a consensus sequence for heat-adapted bacteria (adapted to growth above 60°C). We then compared these consensus sequences using two different strategies. In the comparison strategy, we identified those residues that are highly conserved in both consensus sequences (>60% conserved) but have different identity. For example, this search would reveal a scenario in which a ribosomal protein carries a highly conserved phenylalanine residue in cold-adapted bacteria that is mutated to a highly conserved tyrosine in heat-adapted bacteria. In the second search, we identified those residues that change their conservation in heat-adapted bacteria compared to cold-adapted bacteria (using the 60%). This search would identify a scenario in which a ribosomal protein would have a poorly conserved residue in cold-adapted bacteria (e.g. present in <40% of sequences) but becomes highly conserved (e.g. 100% conserved) in heat-adapted bacteria. The resulting findings of this analysis were combined and mapped on the three-dimensional structures of ribosomal proteins using ChimeraX (43), and the resulting figures are shown in Supplementary Figure S1. To obtain structures of protein bL33 shown in Figure 3B, we downloaded these predicted structures from the AlphaFold repository of predicted protein structures (44,45).

### Assessing frequency of metal-binding residues in ribosomal proteins

To calculate the frequencies of C− and C+ isoforms of ribosomal proteins within each temperature interval, we first split the multiple sequence alignments of each ribosomal protein based on the optimal growth temperature of their corresponding species. This resulted in the creation of eight smaller files per protein, corresponding to temperature bins ranging from 1–10 to 81–90°C. We classified sequences as C+ isoforms if they contained the following patterns: (CXXC)–(CXXC) for proteins uL24, bL28, bL31, bL32 and uS14; (CXXC)–(CXXH) for proteins bS18 and bL36; (CXXC)–(CXXXXC) for protein uS4; and (CXXC)–(CXX[C, D or E]) for protein bL33. If a sequence did not contain any of these patterns, we counted it as a C−. In cases where a species encoded both C− and C+ isoforms of ribosomal proteins, we counted them as C+ because studies conducted in organisms such as *Escherichia coli*, *Bacillus subtilis* and *Mycobacterium tuberculosis* have shown that C+ isoforms of ribosomal proteins are typically expressed under normal physiological conditions, while C− isoforms are expressed during zinc starvation (46–48). The average values of C+ proteins per species in each phylum were then calculated and are shown in Supplementary Table S2.

### Assessing the IVYWREL content within proteins of the minimal gene set or ribosomal proteins

To assess the IVYWREL content of bacterial proteins, we altered the initial approach described in (34) by using protein sequences from the minimal gene set instead of protein sequences of the entire predicted proteomes. In doing so, we wanted to simplify this approach and ensure that we compare a strictly identical set of proteins across different species. Protein sequences were subsequently obtained from the UniProt database, and their YVIWREL content was determined by tallying the cumulative sum of Y, V, I, W, R, E and L residues in all protein sequences of each species and then dividing this sum by the total number of residues in these protein sequences. The YVIWREL content was first calculated for the proteins of the minimal gene set, and then for the ribosomal proteins only. The complete list of proteins used in this analysis, along with their corresponding UniProt IDs, is shown in Supplementary Data 6.

### Mapping bacterial species on the tree of life and detection of distance from LUCA

To illustrate evolutionary variations in ribosomal proteins on the tree of life, we used the tree of life from (49) as the scaffold. This tree was uploaded to the interactive tree of life website (https://itol.embl.de/), where the bacterial branches analyzed in this study were highlighted to measure bacterial distances from the root of tree of life or LUCA and then to create the illustration in Figure 3.

### Comparison of different proxies for estimating optimal growth temperature

To calculate the correlation between optimal growth temperature and GC or U content of bacterial 16S rRNA, we retrieved bacterial 16S rRNA sequences from the RNAcentral database (50). We limited our search to the ‘Silva’ subset of the database, which includes only manually curated sequences. We then refined the dataset by removing plasmid-encoded genes and seemingly truncated rRNA sequences that lacked residues A1492 and A1493 in the decoding site of the ribosome that are indispensable for life (51). Then, to avoid redundancy, we reduced our dataset to one sequence per bacterial species. We accomplished this by selecting a representative sequence for each species, using just one operon per organism (typically, *rrnA*) because rRNA operons in most bacteria were previously reported to have 99.8% or higher levels of sequence similarity (52). This reduced set of sequences was then deposited in Supplementary Data 4 and used to calculate their U content.

To calculate the GC content of helical segments of the 16S rRNA, we first aligned the sequences shown in Supplementary Data 4 using Clustal Omega (41). Next, we have truncated the alignment by leaving only the helical segments from the aligned sequences using the coordinates of helices in the 16S rRNA from *E. coli* (RNAcentral ID URS00000ABFE9_562). Each sequence in the resulting multiple sequence alignment was then annotated by the optimal growth temperature of their corresponding bacterial species, and the alignment was deposited in Supplementary Data 5 and used to calculate the GC content of each sequence.

To calculate the YVIWREL content of amino acids in bacterial proteins, we have retrieved protein sequences corresponding to the minimal set of 61 universally conserved proteins as defined in (53) from the UniProt databank (54) and calculated the sum of their Y, V, I, W, R, E and L divided by the total number of amino acids in these proteins, as initially defined in (34).

To compare the obtained datasets with each other, as shown in Figure 5, we first reduced these datasets to include only the overlapping species between the four correlations, thereby ensuring that each correlation (including the trend line, 95% confidence interval and 95% prediction interval) is calculated for the same set of bacterial species. The corresponding dataset of species shown in Figure 5 was then deposited in Supplementary Data 6.

## RESULTS

### Metal-binding residues are widespread in ribosomal proteins from heat-adapted bacteria

**Figure 1.**
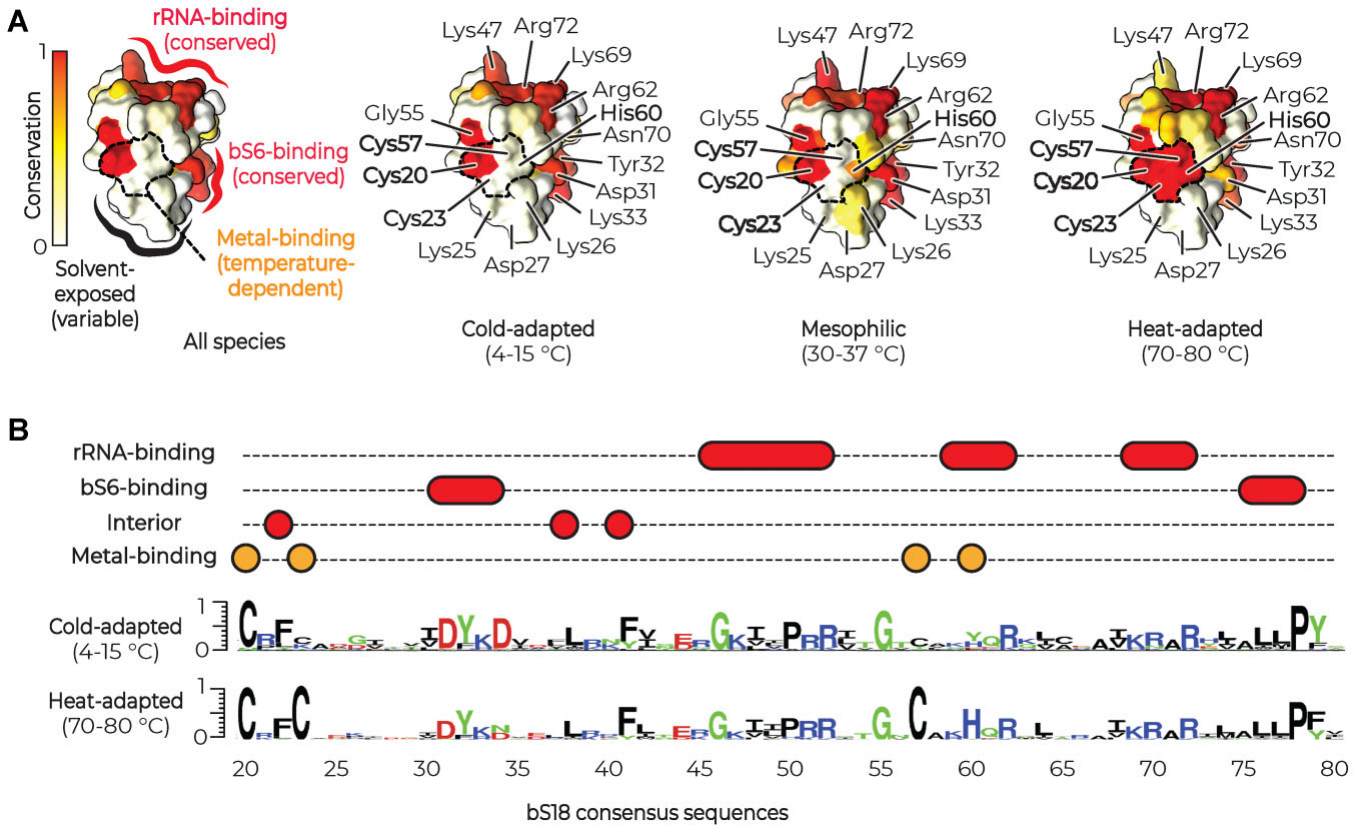
Metal-binding residues are widespread in ribosomal proteins from heat-adapted bacteria. (**A**) The structure of the *E. coli* (PDB ID 4V6F) ribosomal protein bS18 shows how the conservation of protein residues depends on the optimal growth temperature. bS18 possesses three groups of residues. The first group includes residues that remain nearly invariant across all bacteria, regardless of their optimal growth temperature. Most of these mediate bS18 binding to the ribosome. The second group includes residues that remain highly variable across all bacteria, regardless of their optimal growth temperature. These residues are predominantly found on the solvent side of a protein. Only the third and smallest group includes residues whose conservation depends on the optimal growth temperature of bacterial species. These residues remain highly conserved in heat-adapted bacteria but become highly variable in cold-adapted species. In the bS18 structure, these residues coordinate Zn^2+^ ions, which appears to stabilize bS18 folding at high temperatures. (**B**) Conservation ‘logos’ illustrate that the metal-binding residues (Cys23, Cys55 and His60) are the only conserved bS18 residues that distinguish heat-adapted species from non-heat-adapted species. Thus, out of 75 amino acid positions in the structure of bS18, only 3 positions show robust changes in amino acid sequence in a temperature-dependent manner.

We first asked whether metal-binding residues that were found in ribosomal proteins from *T. thermophilus* are present in other heat-adapted species. To test this, we retrieved 24 312 sequences of the ribosomal proteins uS4, uS14, bS18, uL24, bL28, bL31, bL32, bL33 and bL36 from 2021 bacteria (from 825 genera) using our recently established database of organisms with experimentally defined optimal growth temperatures (Supplementary Table S1; see the ‘Materials and Methods’ section) (40). We focused on bacteria because they exist in thermal equilibrium with their environment, and the genome sequences are available for many species with identified optimal growth temperatures. We then aligned these protein sequences and assessed how the identity of each residue in each protein depends on optimal growth temperature (Figure 1 and Supplementary Figure S1).

This analysis revealed that non-thermophilic species possess just a small fraction of highly conserved residues (Figure 1 and Supplementary Figure S1). For example, protein bS18 comprises just 21 conserved residues that mediate bS18 binding to the ribosome and support its folding. In thermophiles, we found the same 21 conserved residues plus 3 additional conserved residues—Cys23, Cys55 and His60—that stabilize bS18 folding by coordinating a Zn^2+^ ion. Similarly, proteins uS4, uS14, uL24, bL28, bL31, bL32, bL33 and bL36 possess just 45 residues that are highly conserved in thermophiles but not in non-thermophilic species (Figure 1 and Supplementary Figure S1). Of these residues, 33 coordinate Zn^2+^ ions (in uS14, uL24, bL28, bL31, bL32, bL33 and bL36) and an iron–sulfur cluster (in uS4). Thus, the metal-binding residues in ribosomal proteins are indeed widespread among thermophilic species, and they represent a very small fraction of residues that distinguish all lineages of thermophilic bacteria from non-thermophilic bacteria.

### Metal-binding residues indicate the limits of the optimal growth temperature

We next asked whether the occurrence of the metal-binding residues correlates with the optimal growth temperature. And, if so, can we use these residues to estimate the optimal temperature for bacterial growth. To test this, we pooled the sequences of ribosomal proteins into eight bins, corresponding to optimal growth temperature intervals from 1–10 to 81–90°C. Then, for each ribosomal protein, we calculated the frequencies of the metal-binding ribosomal proteins in each bin (see the ‘Materials and Methods’ section).

**Figure 2.**
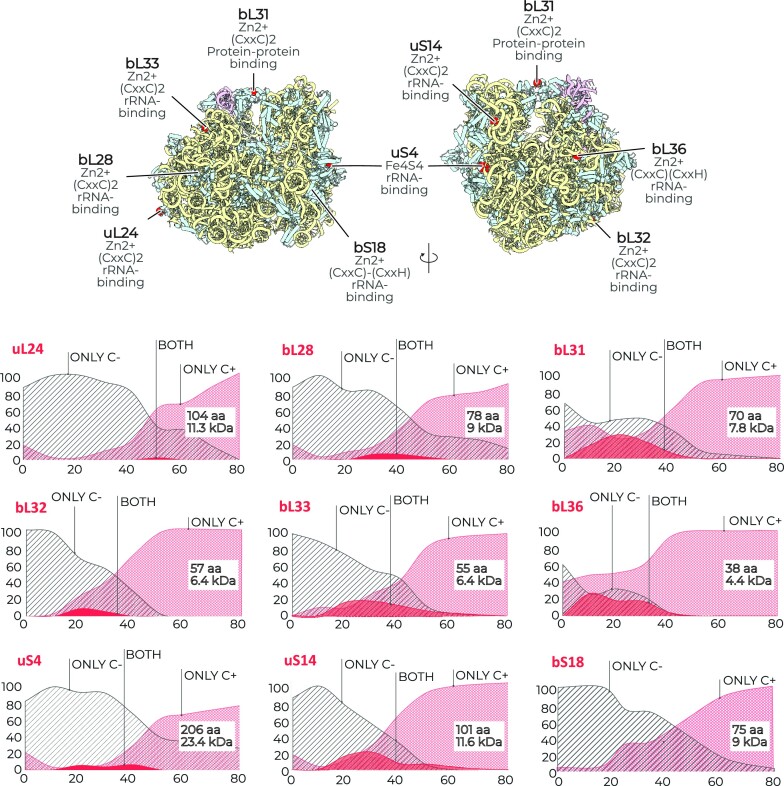
Metal-binding residues indicate the limits of the optimal growth temperature. The structure of bacterial ribosomes from *T. thermophilus* (PDB ID 4Y4Y) illustrating the location of metal-binding sites (CxxC or CxxH) in ribosomal proteins, including eight proteins that bind Zn^2+^ ions and one that binds an iron–sulfur cluster (Fe_4_S_4_). The plots show how the occurrence of metal-binding sites in each of the ribosomal proteins changes with the optimal growth temperatures of bacterial species. Each plot displays three correlations that demonstrate the ratio between three types of bacteria in different temperature intervals: bacteria with a metal-free isoform of a ribosomal protein (‘only C−’), bacteria with a metal-binding isoform of a ribosomal protein (‘only C+’) and bacteria that encode both isoforms of a ribosomal protein (‘both’). Overall, the figure illustrates that upon transition from thermophiles to psychrophiles, the occurrence of metal-binding sites decreases in each ribosomal protein, as evidenced by the reduction in the number of ‘only C−’ organisms compared to ‘only C+’ species.

We found that each ribosomal protein gradually ‘transforms’ from a metal-free isoform (C− isoform) to a metal-binding isoform (C+ isoform) upon transition from cold-adapted to heat-adapted species (Figure 2). For example, in protein bL32, the metal-binding residues are strictly absent in organisms with optimal growth temperatures between 4 and 20°C. Between 21 and 60°C, these residues become more prevalent, and above 60°C, they become strictly conserved.

We also found that the conservation of the metal-binding residues depends on the protein size (Figure 2). For example, in the smallest ribosomal protein (bL36), the metal-binding sites are immutable in all bacteria with optimal growth temperatures of 40°C or more. In larger proteins (uS14, bL31, bL32 and bL33), the metal-binding sites are immutable in bacteria with optimal growth temperatures of 60°C or more. And in even larger proteins (uL24), the metal-binding sites are immutable in bacteria with optimal growth temperatures of 80°C or more. Thus, we found that the metal-binding sites (or their absence) may indicate the lower and upper limits of the optimal growth temperature for bacterial species.

At lower temperatures, however, we observed a few ‘outliers’—cold-adapted organisms that bear C+ ribosomal proteins, which were more prevalent for ribosomal proteins with a smaller molecular weight (Figure 2 and Supplementary Data 2).

### bL33 bears temperature-dependent sequence variations in its metal-binding site

**Figure 3.**
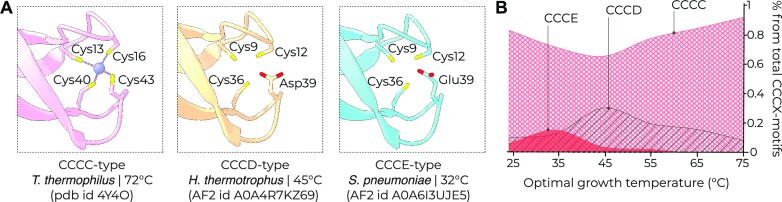
bL33 bears temperature-dependent sequence variations in its metal-binding site. (**A**) Side-by-side comparison of the experimentally defined structure of protein bL33 from *T. thermophilus* and bL33 homologs from species adapted to lower temperatures shows sequence variations in the metal-binding pocket of bL33. (**B**) The plot illustrating the abundance of three apparent metal-binding isoforms of bL33 relative to each other across bacteria with differing optimal growth temperatures.

During our visual assessment of the multiple sequence alignments of ribosomal proteins, we discovered previously uncharacterized sequence variants in the metal-coordinating site of protein bL33. In addition to the previously described CCCC-type sequence variant, in which the metal ion is coordinated by four cysteine residues as was observed in *T. thermophilus* (36), we have identified two additional isoforms: the CCCD-type isoform in which the metal-binding residue Cys39 (in *E. coli* numbering) is replaced with aspartate and the CCCE-type isoform where Cys39 is replaced with glutamate. Both these sequence variants were never characterized experimentally in their ability to coordinate zinc ions in bL33; however, identical sequence motifs have been experimentally reported to coordinate zinc ion in >30 other cellular proteins (55). Also, our analysis of the AlphaFold predictions showed that both CCCD-type and CCCE-type isoforms are predicted to have the same backbone structure of the metal-binding site as in the CCCC-type isoform, suggesting that these bL33 variants can coordinate zinc ions (Figure 3A).

We then asked whether the three isoforms of bL33 are limited to bacteria with a particular range of optimal growth temperatures. To answer this, we calculated the relative abundance of the CCCC, CCCD and CCCE variants of bL33 in bacteria from eight ‘temperature bins’. These bins corresponded to optimal growth temperature intervals ranging from 1–10 to 81–90°C (Figure 3B). We found that the CCCC-type isoform was the most prevalent isoform across the entire range of optimal growth temperatures, whereas the CCCD isoform was most commonly found in moderate thermophiles adapted to growth at 45°C, where ∼30% of the apparent metal-binding bL33 variants were corresponding to this isoform. The CCCE-type isoform also showed temperature-associated variations of its occurrence. It was most frequently observed in bacteria adapted to growth at 32°C, where it accounted for ∼15% of all metal-binding bL33 variants. Thus, the example of bL33 showed that the presence of the metal-binding site is not entirely binary as was initially proposed (and known as ‘two C or not two C’, based on the presence or absence of CXXC motifs in protein sequences) (35). Instead, metal-binding sites in proteins such as bL33 may potentially undergo sequence variations that evolutionary fine-tune their impact on protein thermostability.

### Metal-binding residues are also conserved in ancient branches of non-thermophilic bacteria

**Figure 4.**
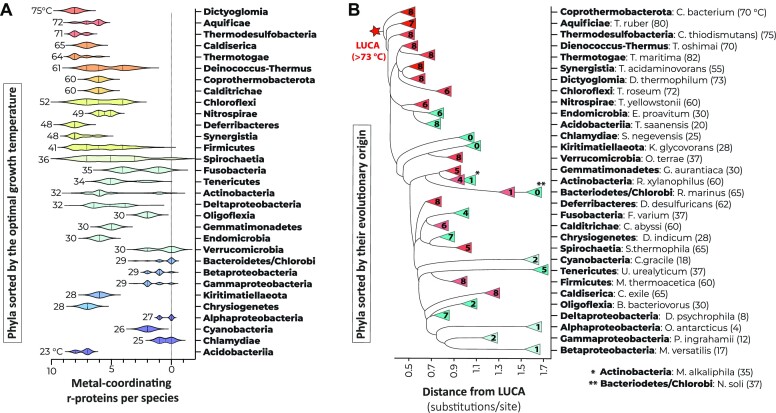
Metal-binding residues are also conserved in ancient branches of non-thermophilic bacteria. (**A**) Bacterial phyla are sorted by their ascending average optimal growth temperature. The labels next to each violin plot (e.g. ‘61’ for Deinococcus-Thermus) indicate an average optimal growth temperature of species from the corresponding phylum. The violin plots are shown to illustrate transition from heat-adapted phyla (top) to cold-adapted phyla (bottom). The panel shows a gradually increasing number of metal-binding ribosomal proteins upon transition from predominantly cold-adapted to predominantly heat-adapted species. However, there are exceptions from this general tendency, such as the phyla of Acidobacteria, Chrysiogenetes and Kiritimatiellaeota. (**B**) A schematic tree of life shows representative species (one or two species per each phylum shown in panel A). The species are organized based on their phylogenetic origin and arranged according to their distance from the bacterial LUCA, as defined in (45). Each species is represented by a triangle that displays the total count of metal-coordinating proteins in the organism (shown as a number inside each triangle). The triangles indicating each species are also colored by the optimal growth temperature, with red indicating thermophilic species and cyan representing cold-adapted species. The actual optimal growth temperature is shown alongside the species name. For instance, the label ‘82’ next to ‘Thermotogae: *T. maritima*’ indicates that *T. maritima* is adapted to growth at 82°C. The panel illustrates that non-thermophilic species with high number of metal-binding ribosomal proteins tend to be located closer to the root of the tree of life. However, the ‘outliers’ (species with ‘anomalously’ few or many C+ ribosomal proteins) do not follow this pattern. For example, δ-proteobacteria possess metal-binding sites in most ribosomal proteins despite being psychrophiles or mesophiles, and they are located as close to LUCA as most thermophiles. Similarly, the heat-adapted bacteria *Rhodotermus marinus* (located at 1.46 substitutions per site distance from the apparent bacterial LUCA) has an optimal growth temperature of 65°C but is atypically remote from the root of the tree compared with other thermophiles and has only one C+ ribosomal protein. These outliers show that the distance from the root of the tree of life correlates with the anomalously high number of metal-binding sites in some cold-adapted bacteria and their anomalously low number in some heat-adapted bacteria.

We then asked whether the organisms with atypically high and low counts of metal-binding ribosomal proteins belong to the same branch of bacteria. To answer this, we calculated the total number of metal-binding ribosomal proteins (C+ proteins) in each species to identify every species with ‘anomalously’ high or low numbers of C+ proteins. We found that, on average, the number of C+ ribosomal proteins gradually increases from psychrophiles (1.5) to thermophiles (8.1) (Figure 4A and Supplementary Table S1). However, 254 bacteria deviated from this tendency by having at least three more or less C+ proteins compared with their average number in a given temperature interval (Figure 4B and Supplementary Data 2).

We then mapped these outliers on the tree of life and found that they belong to different bacterial branches, indicating that C+ ribosomal proteins occasionally occur in evolutionarily distant non-thermophiles (Figure 4A and B, and Supplementary Data 2). Although the outliers belong to different branches of the tree of life, mapping them on the tree of life revealed one common property: their anomalous proximity to LUCA (Figure 3B). Specifically, we found that thermophiles tend to stay closer to LUCA (0.91 substitutions per site in ribosomal proteins) compared to psychrophiles (1.29 substitutions per site), which is consistent with the current theory that life was born in hot environments, with the psychrophiles emerging when the Earth became cooler (56). However, the outliers deviate from this evolutionary tendency (Figure 3B and Supplementary Data 2). For example, the δ-proteobacteria *Desulfotalea psychrophila* has an optimal growth temperature of 8°C but bears eight C+ ribosomal proteins. On the tree of life, this bacterium is located as close to LUCA as an average thermophile (Figure 4B). Overall, this anomalous proximity to LUCA suggests that most outliers could emerge relatively early in the evolution, when the ocean’s temperature was still relatively high.

### Ribosomal proteins can indicate the optimal growth temperature either with higher accuracy or using a lower amount of data

**Figure 5.**
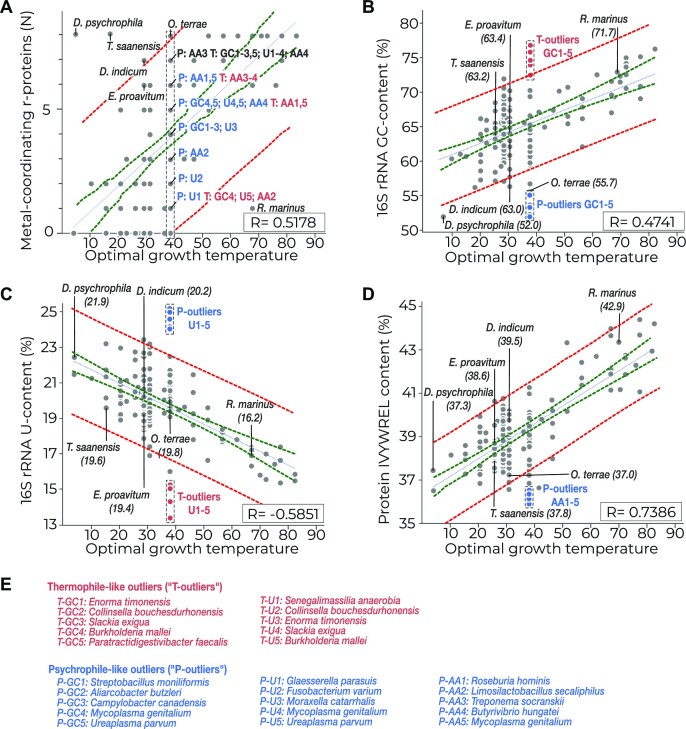
Different proxies of thermal adaptation show comparable predictive power but differ in outliers, illustrating the usefulness of having multiple proxies. Panels (A)–(D) provide a side-by-side comparison of four proxies for estimating the optimal growth temperature for bacterial species, independent of culture. Each of these proxies is based on specific sequence features of proteins and nucleic acids, such as (**A**) the total count of metal-binding ribosomal proteins, (**B**) the GC content in helical segments of the 16S rRNA, (**C**) the U content of the 16S rRNA and (**D**) the IVYWREL content in ribosomal proteins. Each panel is calculated for the same set of bacterial species (represented by dots), with dashed lines indicating the trend line (in blue), the 95% confidence interval (in green) and the 95% prediction interval (in red). The labels in each panel compare two types of outliers. The first set of labels shows species names (e.g. *D. psychrophila*) with the most extreme deviation from the average number of metal-binding sites in ribosomal proteins in their temperature group. The second set of labels designates organisms showing the most extreme deviation from the average value among the organisms adapted to growth at 37°C. For example, the label ‘T-outliers GC1-5’ in panel (B) indicates bacteria with abnormally high GC content of their 16S rRNA compared to other species adapted to 37°C, and the label ‘P-outliers’ indicates bacteria with abnormally low GC content in their 16S rRNA compared to other species adapted to 37°C. (**E**) Species names correspond to the outliers labeled in panels (A)–(D). Overall, the figure shows that most outliers highlighted in plot (A) fall within the 95% prediction interval in plots (B)–(D). Conversely, most outliers highlighted in panels (B)–(D) fall within the 95% prediction interval in panel (A).

We then asked whether the organisms with atypically high and low numbers of metal-binding ribosomal proteins exhibit similar atypical features in other molecules that are used as proxies for thermal adaptation. To answer this, we compared the predicting power of metal-binding sites of ribosomal proteins with three other commonly used proxies, including the GC content in the helical segments of 16S rRNA (20), the U content in the 16S rRNA (17) and the total content of seven amino acids—I, V, Y, W, R, E and L—in cellular proteins (34) (Figure 5A–D).

Using the same dataset of representative bacteria from 181 genera, we found that the optimal growth temperature shows comparable correlations with the presence of metal-binding sites, the 16S rRNA GC content and the 16S rRNA U content, with the Pearson correlation coefficients being *R* = 0.5178, 0.4741 and −0.5851 for these proxies, respectively (Figure 5A and B). This has illustrated a comparable accuracy of using 36 metal-binding residues in a bacterial proteome compared to using the 16S rRNA sequence (Figure 5C). However, consistent with previous studies (34), a significantly higher correlation was observed for the YVIWREL amino acid content in ubiquitous proteins of a cell, which had the Pearson coefficient of 0.7478 (Supplementary Data 6).

Because the IVYWREL content of ubiquitous proteins showed the best correlation with the optimal growth temperature, we recalculated this correlation for sequences of ribosomal proteins alone. In doing so, we wanted to test whether the IVYWREL content of ribosomal proteins can serve as the most accurate proxy among those based on ribosomal genes alone. Our analysis showed that indeed the IVYWREL content of ribosomal proteins showed only a subtly lower correlation with the optimal growth temperature, with the Pierson coefficient of 0.7386 compared to the 0.7478 value obtained for ubiquitous proteins (Figure 5D). Thus, among currently known ribosome-based proxies for thermal adaptation, the IVYWREL content of ribosomal proteins appears to be the most accurate indicator of bacterial thermal adaptation.

### Each ribosome-based proxy of thermal adaptation differs in outliers, illustrating the usefulness of having multiple proxies

Because each of the four analyzed correlations had their own set of outliers, we then highlighted these outliers in each correlation and compared their location in each of our plots (Figure 5A–D). For this purpose, we first examined the GC, U and IVYWREL content values of bacteria that have unusually high or low numbers of metal-binding ribosomal proteins. These outliers included six representative species from the Acidobacteria, Chrysiogenetes, Verrucomicrobiota, Deltaproteobacteria, Endomicrobia and Kiritimatiellaeota phyla (Figure 5B–D). We found that all these outliers fell within the 95% prediction interval in the U and IVYWREL content plots (Figure 5C and D). In the GC content plot, four of the outliers also fell within the 95% prediction interval, and the remaining two outliers—bacteria *Opitutus terrae* and *Desulfotalea psychrophile*—showed an inverse anomaly compared to the count of their metal coordinating sites: both these bacteria are non-thermophiles that have eight metal-coordinating ribosomal proteins, which is typical for heat-adapted species. However, the 16S rRNA GC content in these two bacteria was unusually low and more typical for species adapted to colder temperatures (Figure 5B). Thus, representative bacteria with atypically high and low numbers of metal-coordinating ribosomal proteins either had typical GC, U and IVYWREL content values for bacteria within their temperature range or showed inverse anomaly in their 16S rRNA GC content.

In a complementary analysis, we have identified the total number of metal-binding ribosomal proteins for bacteria with atypically high and low GC, U and IVYWREL content values. For simplicity, we have focused our analysis on the most studied group of species—bacteria adapted to growth at 37°C. Then, for each correlation, we have selected 10 bacteria with the most extreme deviations from the average values of the GC, U or IVYWREL content (Figure 5E). These species included, for example, representative parasitic bacteria from the Firmicutes phylum (e.g. *Mycoplasma* and *Ureaplasma*) that have anomalously low GC content in their 16S rRNA, making these mesophilic organisms resemble cold-adapted bacteria (Figure 5E) (57,58). These species also included, for example, members of the Actinobacteria phylum (e.g. *Rubrobacter* and *Actinobaculum*) that are known for their atypically high GC, making these mesophilic species resemble heat-adapted bacteria (59). Highlighting these outliers on the plot for metal-binding ribosomal proteins, we found all of them residing within the 95% prediction interval, showing the total number of metal-binding proteins close to their average count compared to other species adapted to 37°C (Figure 5A). Collectively, these analyses showed that different proxies for the optimal growth temperature have a certain degree of evolutionary independence from each other, with each proxy having their own set of unique outliers. This observation illustrates the importance of having multiple proxies for culture-independent estimation of the optimal growth temperature.

## DISCUSSION

### The metal-binding sites are adaptive features that are required for bacteria thriving in warm climates

In this study, we have shown that metal-binding sites in ribosomal proteins act as evolutionary adaptations that are necessary for bacteria that flourish at temperatures exceeding 40°C. Conversely, in species adapted to growth below 40°C, these metal-binding sites seem to be dispensable as they gradually disappear in non-thermophilic species as they adapt to lower temperatures and diverge from the root of the tree of life.

This finding is consistent with the current hypothesis that life on Earth has emerged in hot environments, with LUCA being either a thermophile or a hyperthermophile adapted to growth above 75°C (56). This hypothesis stems from the analyses of minerals (60–62) and resurrected enzymes (63–65), suggesting that Earth’s surface has gradually cooled down from 75°C ∼3 billion years ago to 35°C ∼420 million years ago, with a further decrease to 14°C today. If the metal-binding residues pre-existed in LUCA due to their requirement for protein folding at high temperatures, it seems probable that organisms invading colder environments could gradually mutate these residues through neutral variations or through adaptive variations that endow proteins with the flexibility required in colder environments (66–68).

Also, it is important to note that the evolution of the metal-binding sites is very likely a highly complex process that can be influenced by other factors than temperature. One of these possible factors is the independent origin of non-thermophilic species. Previous studies showed that the microbial ability to thrive in cold environments has likely evolved repeatedly and independently throughout the history of life. Some lineages, such as δ-proteobacteria, have likely emerged when the ocean’s temperature varied between 55 and 65°C, whereas other branches of proteobacteria have likely emerged much later, when the ocean’s temperature dropped below 39°C (63). Also, the occasional occurrence of metal-binding residues in non-thermophilic species could stem from alternative mechanisms of cold adaptation. For example, previous studies showed that marked leaps in cold tolerance can be achieved by improving the process of protein folding: the expression of the cold-adapted chaperonins GroEL and GroES enabled *E. coli* growth at −13.7°C, rendering *E. coli* a cold-tolerant organism without a single mutation in its protein sequences (69).

### Compared to rRNA, ribosomal proteins serve as more accurate indicators of bacterial thermal adaptation

In this study, we have also assessed the potential of using sequences of ribosomal proteins to estimate the optimal growth temperature of bacterial species. We found that sequences of ribosomal proteins contain a small fraction of residues that distinguish thermophilic organisms from most non-thermophilic organisms. This finding allowed us to confirm that the metal-binding sites of ribosomal proteins can serve as site-specific, although imperfect, indicators of bacterial thermal adaptation, providing a new tool to infer the limits of optimal growth conditions through sequencing of nature’s most common genes.

Our analysis reveals that the metal-binding sites, as a proxy for thermal adaptation, do not exhibit mathematical precision, as they display a Pearson coefficient of 0.52. However, this level of accuracy surpasses that of the 16S rRNA GC content, which currently serves as the most widely used ribosome-based ‘molecular thermometer’ (31). Consequently, our discovery of mutational hotspots in ribosomal proteins can reduce the amount of data required for experiment-free estimation of optimal growth conditions: instead of relying on the entire 16S rRNA sequence, we can obtain a statistically comparable estimation by using just 36 residues in ribosomal proteins.

Furthermore, because the metal-binding sites in the smallest ribosomal proteins appear to be strictly required for bacteria thriving above 40°C, we can use this property to predict the limits of the optimal growth temperature. For example, if we encounter an uncharacterized organism that lacks the metal-binding residues in bL36, then its optimal growth temperature is likely to be below 40°C because the metal-binding residues in bL36 are strictly conserved in organisms that thrive above 40°C. Conversely, if this organism bears the metal-binding residues in bL32, it is likely that its optimal growth temperature exceeds 20°C because the metal-binding sites in bL32 are strictly absent in bacteria that thrive below 20°C. Thus, mere eight protein residues of the entire cellular proteome, or ∼0.004% of the average bacterial genome, offer substantial insights into the optimal growth temperatures of bacterial species.

At the same time, our analysis of the YVIWREL amino acid content in cellular proteins shows a much higher accuracy as a proxy of thermal adaptation even when the analyzed dataset is reduced to conserved ribosomal proteins. This finding illustrates that variations in the content of full protein sequences remain the most reliable proxy compared to variations in a few specific protein sites.

Collectively, our findings show that ribosomal proteins can serve as more accurate indicators for bacterial thermal adaptation in comparison to the widely used 16S rRNA. By using the metal-binding sites of ribosomal proteins, we can reduce the amount of data without sacrificing prediction accuracy. Alternatively, by using the complete sequences of ribosomal proteins, we can increase prediction accuracy compared to the use of rRNA. This finding may help current attempts to reconstruct the evolutionary history of climate change on our planet and simplify the analysis of uncharacterized species, bypassing the need for laborious, costly and at times impossible experiments.

## Supplementary Material

gkad560_Supplemental_Files

## Data Availability

The data described in this study were deposited in Figshare with the following permanent and citable doi: https://doi.org/10.6084/m9.figshare.22457470.v1.
