# Supplementary material for: Ribosomal proteins can hold a more accurate record of bacterial thermal adaptation compared to rRNA
Source: Nucleic Acids Res. 2023 Jul 3;51(15):8048–59. doi: 10.1093/nar/gkad560 (PMC10450194; doi:10.1093/nar/gkad560)

SUPPLEMENTARY INFORMATION FOR

MANUSCRIPT TITLE

Ribosomal proteins can hold a more accurate record of bacterial thermal adaptation compared to rRNA

AUTHORS
Antonia van den Elzen[1]† Karla Helena-Bueno[1]†, Charlotte R. Brown[1]†, Lewis I. Chan[1] and Sergey V. Melnikov[1,*]

[1] Biosciences Institute, Newcastle University, Newcastle upon Tyne, NE2 4HH, UK
† Joint Authors
* To whom correspondence should be addressed.
Tel: +44(0) 731 149 3559;
Fax: +44 (0) 191 208 6000;
Email: sergey.melnikov@ncl.ac.uk

Content

Supplementary Tables and Figures (in this file)

1. **Supplementary Table S1 |**
   Databases used to collect values of optimal growth temperatures for bacterial species.

2. **Supplementary Table S2 |**
   The occurrence of metal-coordinating ribosomal proteins depends on the optimal growth temperature of bacterial species.

3. **Supplementary Figure S1 |**
   Comparison of consensus sequences for ribosomal proteins from heat-adapted species vs cold-adapted species.

Supplementary Data (available at Figshare following this permanent and citable DOI: https://doi.org/10.6084/m9.figshare.22457470.v1)

1. **Supplementary Data 1 |**
   The list of bacteria and their optimal growth temperatures that were analyzed in this study.

2. **Supplementary Data 2 |**
   Sequences of ribosomal proteins analyzed in this study.

3. **Supplementary Data 3 |**
   Aligned sequences of ribosomal proteins analyzed in this study.

4. **Supplementary Data 4 |**
   Sequences of 16S rRNA annotated by optimal growth temperature and used to calculate their U-content.

5. **Supplementary Data 5 |**
   Sequences of 16S rRNA annotated by optimal growth temperature and used truncated to their helical segments to calculate their GC-content.

6. **Supplementary Data 6 |**
   Comparison of four proxies of thermal adaptation: the total count of metal-coordinating ribosomal proteins, GC- and U-content of 16S rRNA, and YVIWREL-content of proteins.

Table S1. Websites used for web scraping to collect information about the optimal growth temperatures of microbial organisms.

Database	Database Full Name	URL
**ARS (NRRL)**	Agricultural Research Service Culture Collection, National Center for Agricultural Utilization Research, USA	https://nrrl.ncaur.usda.gov/
**ATCC**	American Type Culture Collection, USA	https://www.atcc.org/
**BCCM**	Belgian Coordinated Collections of Microorganisms, Belgium	https://bccm.belspo.be/
**BCRC**	Bioresources Collection and Research Center, Taiwan	https://catalog.bcrc.firdi.org.tw
**CABRI**	Common Access to Biological Resources and Information	http://www.cabri.org/
**CCARM**	Culture Collection of Antibiotics Resistant Microbes, UK	https://www.phe-culturecollections.org.uk
**CCM**	Czech Collection of Microorganisms, the Czech Republic	https://www.sci.muni.cz/ccm/
**CCUG**	Culture Collection, University of Goteborg, Sweden	https://www.ccug.se/
**CECT**	Spanish Type Culture Collection, Spain	https://www.uv.es/cect/
**CICC**	China Center of Industrial Culture Collection	http://english.china-cicc.org
**CIP**	Center for Biological Resources of the Institute Pasteur, France	https://catalogue-crbip.pasteur.fr
**DSMZ**	German Collection of Microorganisms and Cell Cultures GmbH, Germany	https://www.dsmz.de
**GRIN**	Agricultural Genetic Resources Information Center, USA	https://www.ars-grin.gov
**HAMBI**	Culture Collection of Department of Applied Chemistry and Microbiology, University of Helsinki, Finland	https://kotka.luomus.fi/culture/bac
**JCM**	Japan Collection of Microorganisms (RIKEN Bioresource Center), Japan	https://jcm.brc.riken.jp/en/ordering_e
**KEMB**	Korea Environmental Microorganisms Bank, South Korea	https://kemb.or.kr
**NBIMCC**	National Bank for Industrial Microorganisms and Cell Cultures, Bulgaria	https://www.nbimcc.org/en/about.htm
**NBRC**	NITE Biological Resource Center, Japan	https://www.nite.go.jp
**NCIMB**	National Collections of Industrial, Food and Marine Bacteria, UK	https://www.ncimb.com/
**NCMA**	National Center for Marine Algae and Microbiota (NCMA), USA	https://ncma.bigelow.org/
**NCTC**	National Collection of Type Cultures, UK	https://www.phe-culturecollections.org.uk/
**NIES**	National Institute for Environmental Studies, Japan	https://mcc.nies.go.jp/
**VKM**	All-Russian Collection of Microorganisms, Russia	http://www.vkm.ru

Table S2. Websites used for web scraping to collect information about the optimal growth temperatures of microbial organisms.

Bacteria lineage	Average optimal growth temperature	Average number of metal-coordinating r-proteins
Acidobacteria	23.3	7.3
PVC group;Chlamydiae	25	0.5
Terrabacteria group;Cyanobacteria/Melainabacteria group	26	2
PVC group;Planctomycetes	27.4	4.4
Proteobacteria;Alphaproteobacteria	27.7	0.4
Chrysiogenetes;Chrysiogenetes	28	7
PVC group;Kiritimatiellaeota	28	6
Proteobacteria;Gammaproteobacteria	28.6	1.5
Proteobacteria;Betaproteobacteria	28.9	1.1
FCB group;Bacteroidetes/Chlorobi group	29.4	0.5
PVC group;Verrucomicrobia	30	0.6
Elusimicrobia;Endomicrobia	30	6
FCB group;Gemmatimonadetes	30	5
Proteobacteria;Oligoflexia	30	2
Terrabacteria group;Actinobacteria	31.7	4.9
Proteobacteria;delta/epsilon subdivisions	31.9	6
Terrabacteria group;Tenericutes	33.5	4.2
Fusobacteriia	35.2	2.5
Spirochaetia	36.4	4.8
Terrabacteria group;Firmicutes	40.7	5.6
Synergistia	47.8	7.4
Deferribacteres	48	8
Nitrospira	48.5	5.5
Terrabacteria group;Chloroflexi	51.5	5.8
Calditrichaeota;Calditrichae	60	6
Terrabacteria group;Coprothermobacterota	60	6
Terrabacteria group;Deinococcus-Thermus	60.7	5.2
Thermotogae	64.6	7.5
Caldisericia	65	7
Thermodesulfobacteria	71.6	7.6
Aquificae	72.2	6.5
Dictyoglomia	75	8

Figure S1. This figure compares two consensus sequences of each metal-coordinating ribosomal protein, with one of these consensus sequences corresponding to cold-adapted bacteria (on the left) and another one corresponding to heat-adapted bacteria (on the right). The red boxes highlight the location of the metal-coordinating residues in each ribosomal protein.

uL24

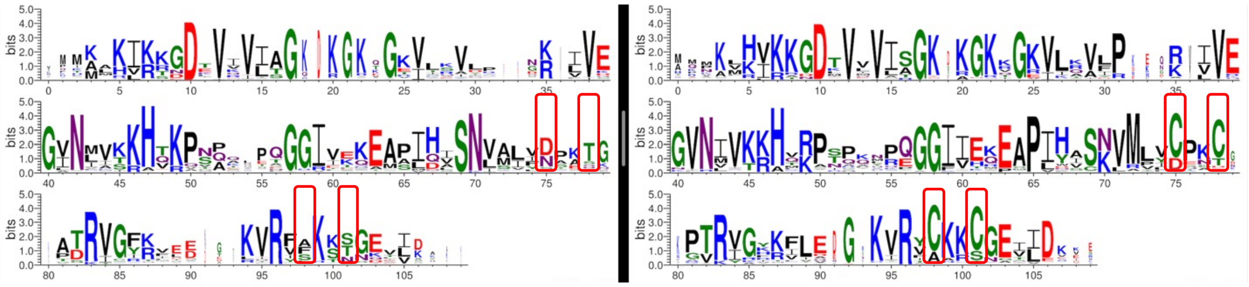

bL28

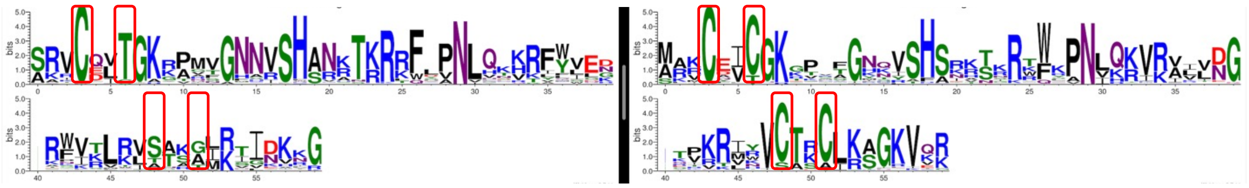

bL31

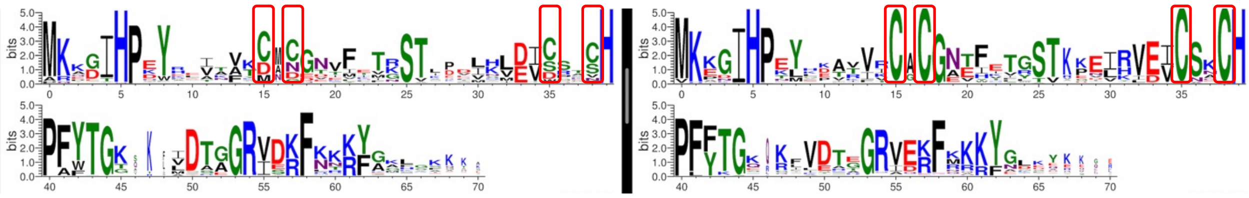

bL32

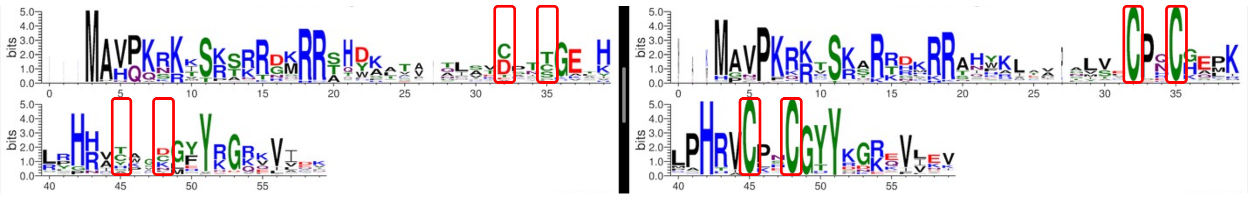

bL33

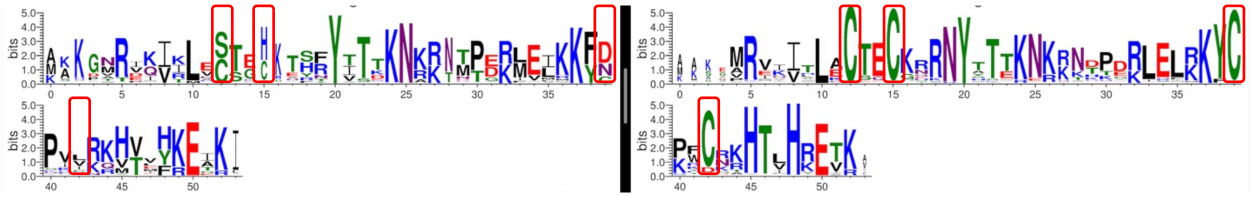

bL36

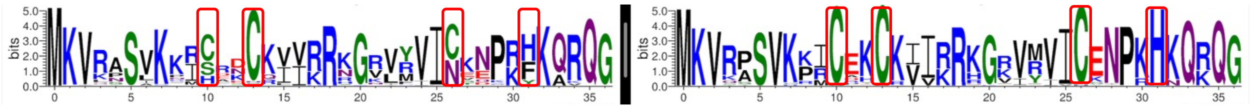

uS4

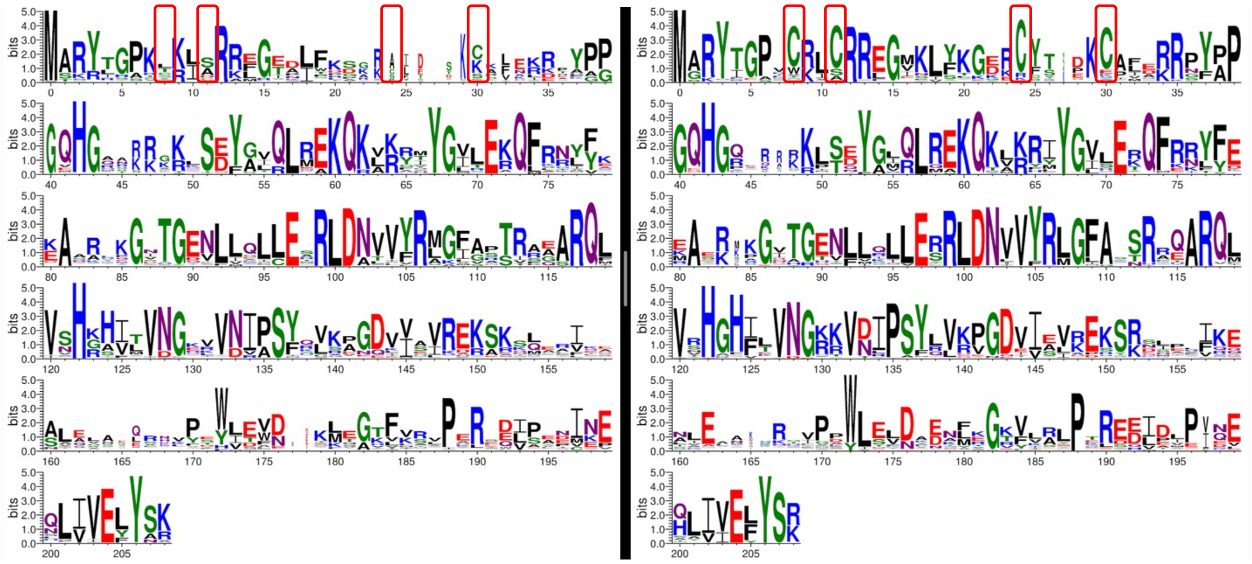

bS18

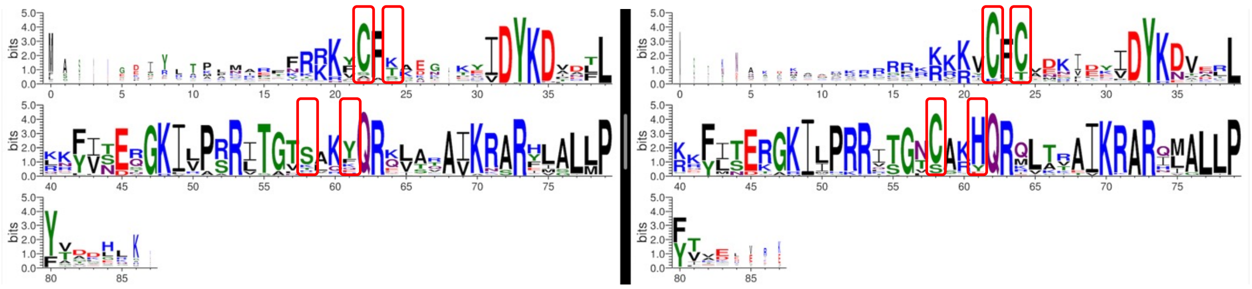

Supplement: gkad560_Supplemental_Files [file gkad560_supplemental_files.zip › NAR-02895-R-2022-R2-SI.pdf]
